# Should population-based research steer individual health decisions?

**DOI:** 10.18632/aging.102446

**Published:** 2019-11-04

**Authors:** Kai O. Hensel, Michelle R. Longmire, Jöran Köchling

**Affiliations:** 1 Cambridge University Hospitals NHS Foundation Trust, Department of Paediatrics, Addenbrooke’s Hospital, Cambridge Biomedical Campus, Cambridge, UK; 2 Witten/Herdecke University, Center for Clinical & Translational Research (CCTR), Department of Paediatrics, Faculty of Health, Witten, Germany; 3 Medable Inc., Palo Alto, CA 94301, USA

**Keywords:** hangover, beer, wine, myth, personalised trials

Alcohol consumption is a significant avoidable global health risk [1]. While long-term sequelae are well-studied, acute alcohol-induced hangover is a major, but understudied, global hazard and a substantial socioeconomic burden [2]. Surprisingly, there are no sound pathophysiological hangover models or effective treatments. However, related old folk wisdoms exist in numerous languages and variations, e.g. “*Beer before wine and you’ll feel fine, wine before beer and you’ll feel queer*”. To evaluate whether this time-honored concept may serve as a basis to systematically reduce the hangover burden, we undertook a randomized controlled multi-arm matched-triplet crossover interventional trial [3].

In brief, participants consumed – under strictly controlled experimental conditions – either beer followed by wine, or vice versa, or only one of these beverages. On a second occasion (crossover, ≥ 1 week later), all participants were switched to the respective opposite drinking regimen. Primary endpoint was the volunteers’ hangover intensity on the subsequent day. Consumed alcohol quantity was guided according to maximal breath alcohol concentration; caloric intake and water consumption prior to the interventions and afterwards were controlled.

Interestingly, on overall comparison neither type nor order of consumed alcoholic drinks significantly affected next-day’s hangover severity. Multivariate regression analyses failed to identify solid objective (epidemiological, laboratory biochemistry, etc.) predictors for hangover intensity, while subjective red flag symptoms such as perceived drunkenness and vomiting were associated with increased hangover severity. One could hence conclude, that it is rather the quantity than the mixing of alcohol beverages which leads to a more intense hangover.

**Figure 1:**
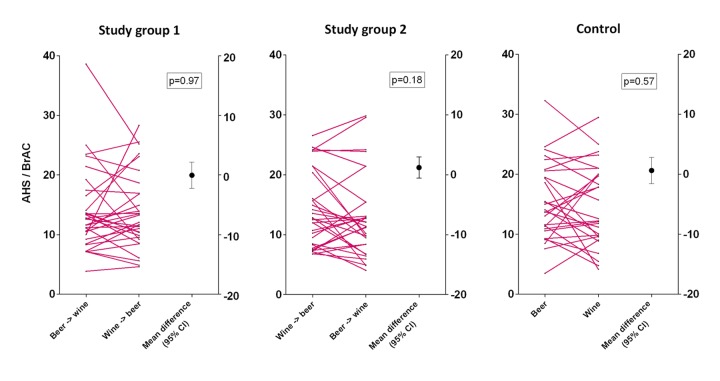
This illustration depicts mean vs. individual differences for hangover intensity in a randomized controlled crossover trial. Hangover severity is expressed as the ratio of acute hangover scale rating (AHS) and maximal breath alcohol concentration (BrAC) in accordance to type and order of alcoholic beverage consumption. For all groups, AHS/BrAC ratios are given for each individual on both study day participations (magenta), e.g. study group 1 on study day 1: beer first, then wine; vs. study day 2 (crossover for the same individual): wine first, then beer, etc. Mean differences and 95% confidence intervals are shown on the right (black). Remarkably, while mean differences are small with narrow 95% CI, a considerable number of individuals demonstrated a strikingly different hangover response when comparing the two study participations. Because this individual effect is bi-directional, the result is an overall small mean difference for the cohort as a whole [3].

A myth debunked? Importantly, a closer look at the data set reveals a crucial aspect that only comes to light when datapoints are examined at an individual level: While mean differences of order (i.e. beer first vs. wine first) and type (beer only or wine only) are near zero, there is a subset (≈20% of study subjects) for which there was in fact a substantial measurable intra-individual hangover intensity difference (*Figure 1*). In other words, the main study conclusion does not apply to approximately one in five study participants.

So what does this mean for the study? And to what extent does this phenomenon apply to other studies and their findings? The first approach to tackle this is the “power-in-numbers” philosophy, which argues that larger cohorts and more trials may yield the correct answer. In contrast, Glasziou et al. have analyzed the reliability of single trials in settings where meta-analyses are not available – as in this scenario. Interestingly, most precise trials revealed similar estimates of effects to meta-analyses, while “negative” results were found to be less reliable [4]. Moreover, the question whether size matters remains unanswered also when it comes to minimizing bias in clinical trials [5]. After all, a larger trial is only superior to a smaller one, if the studied hypothesis can be generalized and the included participants are in fact comparable. To establish this, we utilized a thorough matched-triplet study design, in which participants were only randomized into the study groups if they could be matched into triplets of similar epidemiological “hangover-relevant” characteristics (such as age, BMI, drinking experience, etc.). Figure 1 shows, that either order and type of alcoholic beverages have a completely random effect (null hypothesis), or there are cohort subsets that in fact respond differently to the intervention and the underlying pattern yet remains to be illuminated.

This leads to another approach: should trials focus away from the population towards the individual, or aim to include both? The concept of “population health” and “individual health” is a relatively well-established and rather fashionable dichotomy [6]. However, most researchers do not investigate both these aspects in the same study. Clearly, the relationship between population (or here: study cohort) and individual health is complex, often context-dependent and probably at times dynamic [7]. However, an important question is often missed when interpreting trial results: What if a research hypothesis is not either 100% correct or wrong for the entire cohort, but true for some and wrong for others? Is a yes-or-no question the best approach to tackle a problem of unknown complexity?

In times of personalized medicine, genotyping and epigenetic landscape mapping, clear yes-or-no answers are increasingly rarer as trial cohorts turn out to be diverse. This results in an increased complexity of research findings. In a scenario where a research hypothesis only applies for a certain fraction of study participants, the only way to avoid a false generalization is to identify relevant aspects that distinguish the (often unintentionally present) study subgroups. This necessarily includes the collection of more data than we usually would in traditional trials. Decreasing costs for (epi-)genetic testing and the rapidly expanding possibilities of mobile digital technologies such as smartphone apps and wearable sensors will likely play a major role in this development in the future. Finally, the extreme opposite of a large population based approach to predict individual outcomes is the concept of the n-of-1 trial – the ultimate strategy for individualizing medicine [8]. After all, whether the research focus should be rather broad or deep will always be context (and resource) dependent. One thing is certain, future clinical trial design will become more challenging if we want to embrace technical innovations and open our eyes to the ever-increasing complexity out there. Cheers!
